# Rectus sheath haematoma following exercise testing: a case report

**DOI:** 10.4076/1752-1947-3-9000

**Published:** 2009-08-24

**Authors:** Laszlo Barna, Imre Toth, Erzsebet Kovacs, Eszter Krizso

**Affiliations:** 1 Department of Cardiology and Internal Medicine, Borsod County Hospital and Teaching Hospital, Miskolc, Szentpéteri kapu, Hungary; 2 Department of Surgery, Semmelweis Teaching Hospital, Miskolc, Csabai kapu, Hungary; 3 Department of Radiology, Borsod County Hospital and Teaching Hospital, Miskolc, Szentpéteri kapu, Hungary; 4 AA-MED Ltd., CT Department, Miskolc, Csabai kapu, Hungary

## Abstract

**Introduction:**

Exercise testing is a safe diagnostic procedure which is widely used in the evaluation of patients suspected of having coronary heart disease or for the assessment of the prognosis in patients with established disease. Its complications are mainly cardiac disorders. Here, we report a rectus sheath haematoma as a complication of this procedure in a patient with acute coronary syndrome. To our knowledge, this is the first case report of rectus sheath haematoma in association with exercise testing.

**Case presentation:**

A 72-year-old Caucasian woman was admitted for acute coronary syndrome. She received conservative treatment including low molecular weight heparin and anti-platelet agents. On the fifth day of her hospital stay, she underwent an exercise test, where no ischaemic response occurred. Several hours later, she experienced pain in the left side of her abdomen. Subsequent investigations revealed a rectus sheath haematoma. The patient underwent surgical haematoma evacuation. A few days later, re-operation was performed for recurrent bleeding in the abdominal wall. The patient had several characteristics known to increase the risk of bleeding during treatment for acute coronary syndrome.

**Conclusion:**

Awareness of this possible consequence of exercise testing is important for preventing and treating it correctly. For prevention, an assessment of the bleeding risk of the individual patient is necessary before the test, and excessive anticoagulation must be avoided.

## Introduction

Exercise testing is a safe diagnostic procedure that is widely used in the evaluation of patients suspected of having coronary heart disease or for prognostic purposes in patients with established disease. It plays an important role in the assessment of patients treated for acute coronary syndrome if they are initially stratified in the low-risk group. Complications of exercise testing are mainly cardiac disorders such as arrhythmias and coronary events. Although significant bleeding is an extremely rare consequence of this procedure, one case of bleeding complication has been reported in the literature [1]. On the other hand, bleeding is not an infrequent event during treatment of acute coronary syndrome because of the administration of potent anticoagulants and anti-platelet agents.

We report a rectus sheath haematoma as a complication of exercise testing in a patient with acute coronary syndrome.

## Case presentation

A 72-year-old Caucasian woman presented with squeezing chest pain. The pain had started 30 minutes previously and was associated with nausea, vertigo, shortness of breath and weakness. Her history was significant for hypertension and coronary artery disease. Two years before admission, she had undergone coronary angiography because of angina, which revealed three-vessel coronary disease. The examination was followed by repeated angioplasty with stent implantation. A drug-eluting stent had also been applied. The first intervention had been complicated by a haematoma at the femoral puncture site.

On physical examination, the patient had a blood pressure of 130/60 mmHg and a heart rate of 65 beats per minute. The lung and heart sounds were clear on auscultation. Her weight was 60 kg, her height was 156 cm and her body mass index was 24.65 kg/m^2^. An electrocardiogram showed normal sinus rhythm without signs of myocardial ischaemia. Laboratory testing was significant for haemoglobin at 11.4 g/dL, blood urea nitrogen at 10.45 mmol/L and creatinin at 131 μmol/L. The creatinin clearance calculated with the Cockroft-Gault equation was 26.47 mL per minute. There was no elevation of cardiac troponin-I and creatine phosphokinase-MB isoenzyme levels.

After admission, her symptoms soon subsided. Repeated electrocardiography did not show signs of ischaemia, and the troponin-I value remained in the normal range. A diagnosis of unstable angina was established. The patient received early conservative treatment with the following pharmacotherapy: 2 × 60 mg enoxaparin subcutaneously, 75 mg clopidogrel, 100 mg aspirin, 50 mg metoprolol, 20 mg atorvastatin, 0.2 mg per hour transdermal nitroglycerin, 80 mg valsartan and 12.5 mg hydrochlorothiazide.

**Figure 1:**
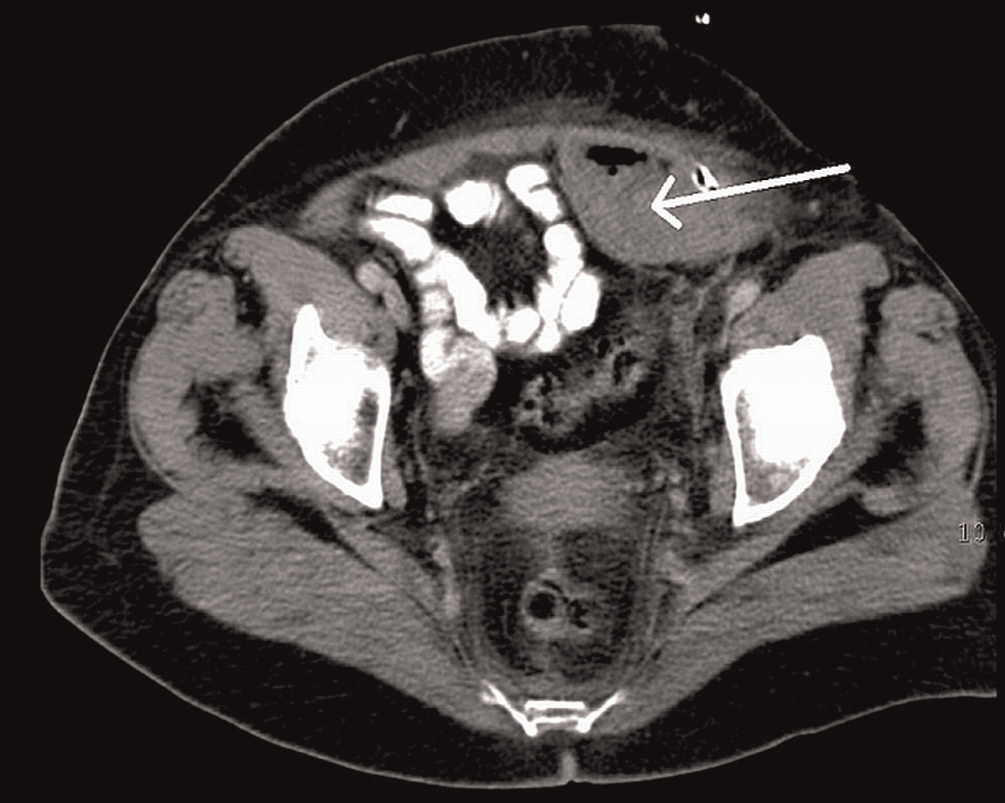
**Computed tomography scan showing haematoma in the left rectus sheath (arrow)**.

On the fifth day of her hospital stay, an exercise stress test was performed on a bicycle ergometer where she reached 3.9 metabolic equivalent and no ischaemic response occurred. A few hours later, she experienced pain in the left side of her abdomen. On examination, a palpable, growing tender mass was detected in the lower left quadrant. An abdominal ultrasound scan demonstrated a rectus sheath haematoma with a depth of 20 mm and a width of 40 mm. The upper margin of the haematoma was at the umbilicus, the lower reached the pubic bone. As continued haemorrhage was suspected, the patient underwent surgical exploration with haematoma evacuation, and received a blood transfusion. She remained on a reduced dose of enoxaparin (1 × 60 mg). Since she was febrile and showed a decreasing haemoglobin level, on the 9th postoperative day a computed tomography (CT) scan was performed. This showed a recurrence of the rectus sheath haematoma (Figure 1) and its extension in the abdominal wall. A second haematoma evacuation was performed. Subsequently, no recurrence of bleeding was observed, but the patient developed an ascending superficial thrombophlebitis on the left lower limb, which was treated with ligature. She was finally discharged in good health.

## Discussion

Rectus sheath haematoma is an unusual cause of abdominal pain. It is frequently misdiagnosed and confounded with other intra-abdominal pathologies. The underlying mechanism is the rupture of epigastric vessels. The lower quadrants are most commonly involved. Rectus sheath haematoma may be related to trauma of the abdominal wall, complications of surgery or subcutaneous injection of different agents in the abdominal wall. However, it frequently occurs without obvious trauma, such as when coughing. In this situation, the intense contraction of the rectus muscle may cause tearing of branches of epigastric vessels. Other conditions predisposing to rectus sheath haematoma include arteriosclerotic disease and anticoagulant treatment [2,3]. Renal disease may also predispose to this bleeding event, since it is frequently reported in patients with rectus sheath haematoma. In a series of cases where low molecular weight heparin (LMWH) was used as an anticoagulant, five out of six patients had impaired renal function [4]. A possible explanation is that LMWH is partly eliminated by the renal route and administration of the usual doses may result in accumulation and excessive anticoagulation in these patients.

Abdominal ultrasonography and CT scans provide useful information for the differential diagnosis and can give a precise description of haematoma localisation.

If diagnosis is unequivocal and the bleeding does not continue, conservative management is preferable. Other therapeutic opportunities include surgical intervention and trans-catheter embolisation [2,5]. If the patient is treated with anticoagulants, these should be stopped and, if possible, antidote or coagulant factor replacement can be applied.

In our patient, several risk factors for bleeding during treatment for acute coronary syndrome were present: a history of bleeding, female gender, impaired renal function and co-administration of aspirin and clopidogrel [6]. The dose of enoxaparin was not properly adjusted to her renal function, which might have resulted in excessive anticoagulation. We presume the exercise testing will have caused strain of the abdominal wall, which, in the presence of the predisposing factors, led to the bleeding in the rectus sheath. The mechanism is similar to the case of cough. The time between the exercise test and the first complaints of the patient was about 4 hours. Despite this delay, we believe exercise testing was the triggering event since during this time no other physical trauma occurred. The delay can be explained by the moderate intensity of bleeding and the decreased pain perception of the older patient.

The diagnosis was obvious after the first ultrasound examination. Surgical treatment seemed to be the proper therapeutic option because continued bleeding was suspected. Surgery, however, was followed by repeated exploration and lower extremity thrombophlebitis. These complications might possibly have been avoided with a more conservative initial approach.

To our knowledge, this is the first case report of rectus sheath haematoma in association with exercise testing.

## Conclusions

Our report draws attention to an unusual complication of exercise testing. Awareness of this possible consequence of this widely used procedure is important in order to prevent and treat it correctly. For prevention, the assessment of bleeding risk of the individual patient is necessary before the test, and excessive anticoagulation must be avoided. The dose of LMWH must be calculated taking account of the patient's body weight and renal function.

## Consent

Written informed consent was obtained from the patient for publication of this case report and accompanying image. A copy of the written consent is available for review by the Editor-in-Chief of this journal.

## Competing interest

The authors declare that they have no competing interests.

## Authors' contributions

LB made substantial contributions to conception and design, interpretation of data and formulation of the manuscript. IT interpreted the surgical intervention. E. Kovacs performed the sonographic examination and interpreted it. E. Krizso performed the CT examination and interpreted it. All authors read and approved the final manuscript.
